# The Responses of Tissues from the Brain, Heart, Kidney, and Liver to Resuscitation following Prolonged Cardiac Arrest by Examining Mitochondrial Respiration in Rats

**DOI:** 10.1155/2016/7463407

**Published:** 2015-12-07

**Authors:** Junhwan Kim, José Paul Perales Villarroel, Wei Zhang, Tai Yin, Koichiro Shinozaki, Angela Hong, Joshua W. Lampe, Lance B. Becker

**Affiliations:** ^1^Center for Resuscitation Science, Department of Emergency Medicine, University of Pennsylvania, Philadelphia, PA 19104, USA; ^2^College of Liberal Arts and Sciences, Villanova University, Villanova, PA 19085, USA

## Abstract

Cardiac arrest induces whole-body ischemia, which causes damage to multiple organs. Understanding how each organ responds to ischemia/reperfusion is important to develop better resuscitation strategies. Because direct measurement of organ function is not practicable in most animal models, we attempt to use mitochondrial respiration to test efficacy of resuscitation on the brain, heart, kidney, and liver following prolonged cardiac arrest. Male Sprague-Dawley rats are subjected to asphyxia-induced cardiac arrest for 30 min or 45 min, or 30 min cardiac arrest followed by 60 min cardiopulmonary bypass resuscitation. Mitochondria are isolated from brain, heart, kidney, and liver tissues and examined for respiration activity. Following cardiac arrest, a time-dependent decrease in state-3 respiration is observed in mitochondria from all four tissues. Following 60 min resuscitation, the respiration activity of brain mitochondria varies greatly in different animals. The activity after resuscitation remains the same in heart mitochondria and significantly increases in kidney and liver mitochondria. The result shows that inhibition of state-3 respiration is a good marker to evaluate the efficacy of resuscitation for each organ. The resulting state-3 respiration of brain and heart mitochondria following resuscitation reenforces the need for developing better strategies to resuscitate these critical organs following prolonged cardiac arrest.

## 1. Introduction

Cardiac arrest (CA) induces whole-body ischemia, which causes damage to multiple organs, including the brain, heart, kidney, and liver [1]. The damage to these vital organs is responsible for the high mortality and morbidity of patients with CA. However, how each organ responds to ischemia/reperfusion and further contributes to the mortality of patients is not understood. This information is critical to develop better resuscitation strategies to enhance the survival rate of patients, which has not improved over the last few decades.

The exact pathology of ischemia/reperfusion injury remains poorly described in CA. In particular, the fact that additional damage following resuscitation, when observed, is from reperfusion injury or poor perfusion is not clear [2]. The interpretation is more complicated by the fact that damage to one tissue can influence the severity of damage or recovery of other tissues [3]. This complication may be overcome by monitoring the function of each organ following ischemia and resuscitation. However, direct measurement of organ function is not practicable in most animal models of CA.

Ischemia causes various alterations in the mitochondrial electron transport chain [4, 5]. Particularly, respirational defects are the most commonly observed ischemic alteration [6–8] and rescuing mitochondrial function is the key to successful resuscitation [9]. These reports suggest that mitochondrial respiration is a good indicator to examine the efficacy of resuscitation protocols on individual tissues. To further explore this hypothesis, we subjected rats to asphyxia-induced CA and cardiopulmonary bypass (CPB) resuscitation. Mitochondria were isolated from brain, heart, kidney, and liver tissues and examined for respiration activity. The CPB system is advantageous when studying the effect of reperfusion because mechanically supported circulation provides a relatively consistent blood flow compared to conventional CPR [10, 11], in which the blood flow relies on the recovery of heart function.

We first compared mitochondrial state-3 (ADP-dependent), state-4 (ADP-limited), and uncoupled respiration activities between 30 min CA and 45 min CA to understand how the activities change over time under ischemic conditions. The comparison also tests whether the respiration activity observed following 30 min CA has reached its minimum or whether an additional decrease is possible. We then compared 30 min CA to 30 min CA followed by 60 min CPB resuscitation. The results show that state-3 respiration is a good indicator to provide insights into tissue-specific responses of these vital organs to CA and resuscitation.

## 2. Materials and Methods

### 2.1. Animals

The experimental protocol was approved by the Institutional Animal Care and Use Committee of the University of Pennsylvania. Adult male Sprague-Dawley rats (weight 465–530 g), housed in a rodent facility with unrestricted access to food and water, were used for the study. The numbers of animals were 8 for control, 7 for 30 min CA, 6 for 45 min CA, and 8 for 30 min CA plus 60 min CPB resuscitation. All data were presented as mean ± standard deviation. Group comparisons were made with one-way analysis of variance and Bonferroni's test was used as a* post hoc* test; a *p* value < 0.05 was considered statistically significant.

### 2.2. Asphyxia-Induced Cardiac Arrest and Cardiopulmonary Bypass

**Scheme 1:**
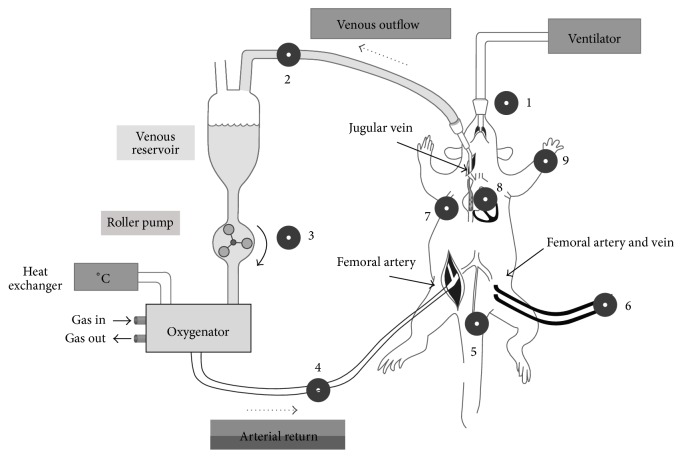
Diagram showing surgical procedure of asphyxial CA and CPB resuscitation. 1. Capnograph. 2. Oxygen saturation and hematocrit (Critline). 3. Pump. 4. Temperature, PO_2_, and system pressure of arterial return. 5. Rectal temperature. 6. Arterial and central venous blood pressure, ABG. 7. ECG. 8. Esophageal temperature. 9. Pulse oximetry.

The detailed procedures were published elsewhere [12] and the experimental setup of the model is shown in Scheme 1. Briefly, rats were anesthetized with 4% isoflurane and ventilated with an orotracheally intubated catheter with 2% isoflurane. Heparin (150 U) and vecuronium (1 mg) were administered through the left femoral vein. Asphyxia was induced by stopping the ventilator and isoflurane was discontinued thereafter. Within 3 min of asphyxia, mean arterial pressure fell below 20 mmHg, our definition of CA [12]. Following 30 min CA, resuscitation was started with the initiation of CPB flow and resumption of ventilation. The CPB flow rate was adjusted to meet venous outflow. At the end of experiment, rats were sacrificed by decapitation to collect the tissues. Control rats were decapitated 7 min after administration of isoflurane.

### 2.3. Mitochondria Isolation

#### 2.3.1. General Procedure

All procedures were performed at 4°C. The mitochondria isolation protocol was developed by modification of the method used for muscle mitochondria [13]. Each brain, heart, liver, and kidney was immediately placed in mitochondrial isolation buffer (MESH) composed of 210 mM mannitol, 70 mM sucrose, 10 mM Hepes, and 0.2 mM EGTA, at pH 7.3. The tissues were trimmed in MESH buffer to remove spinal cord, extraventricular tissue, and fats, blot-dried on filter paper, weighed, and placed in MESH buffer freshly supplemented with 0.2% w/v fatty acid-free BSA. The tissues were homogenized with a teflon/glass motor-driven homogenizer (Glas-Col LLC., Terre Haute, IN). A Beckman model J-30-1 centrifuge (JA-30.50 rotor) was used with low speed centrifugation at 5600 ×g for 1 min and high speed centrifugation at 10000 ×g for 6 min. Mitochondria concentrations were determined by the BCA assay and expressed as mg mitochondrial protein/g tissue.

#### 2.3.2. Brain Mitochondria Isolation

Minced brain tissue in MESH-BSA (10 mL/g tissue) was homogenized for 8 strokes at the setting of 40. The homogenates were centrifuged at low speed and the supernatant was poured into a polycarbonate tube. The pellet was homogenized and centrifuged as above and the pooled supernatant was centrifuged at high speed. The supernatant was poured out gently until synaptosomes layer reached the top. The remaining loose pellet was suspended with 20 mL of 12.5% Percoll in MESH (v:v) and centrifuged at high speed. The supernatant was gently decanted with pipets without disturbing the mitochondria pellet (usually ~200 *μ*L buffer remains). Finally, the pellet was resuspended in 20 mL of MESH buffer and centrifuged at high speed. The mitochondria pellet was suspended in 0.05 mL MESH per g tissue to yield ~20 mg/mL protein concentration.

#### 2.3.3. Heart Mitochondria Isolation

Minced heart tissue in MESH-BSA (15 mL/g tissue) was homogenized for 12 strokes at the setting of 30 [13]. The homogenates were centrifuged at low speed and the supernatant was saved. The homogenization of the pellet and centrifugation were repeated two more times (the third homogenization was in 10 mL/g tissue). The pooled supernatant was centrifuged at high speed. The pellet was resuspended in 20 mL of MESH buffer and centrifuged at high speed again. The mitochondria pellet was suspended in 0.5 mL MESH per g tissue to yield ~25 mg/mL protein concentration.

#### 2.3.4. Kidney and Liver Mitochondria Isolation

Minced kidney or liver tissue in MESH-BSA (10 mL/g tissue) was homogenized for 3 strokes at the setting of 30. The homogenates were centrifuged at low speed and the supernatant was centrifuged at high speed. The pellet was resuspended in 20 mL of MESH buffer and centrifuged at high speed. The mitochondria pellet was suspended in 0.4 mL MESH per g tissue to yield ~30 mg/mL protein concentration for kidney and ~45 mg/mL for liver mitochondria.

### 2.4. Oxidative Phosphorylation

Oxygen consumption was measured using a Strathkelvin oxygen electrode (30°C) in a buffer containing 80 mM KCl, 50 mM MOPS, 1 mM EGTA, 5 mM KH_2_PO_4_, and 1 mg defatted BSA/mL at pH 7.4 [14, 15]. State-3, state-4, and uncoupled respirations were measured in 150 *μ*L of the mitochondrial suspension (0.5 mg/mL) using glutamate + malate as substrates. Respiration rates were expressed as nanoatoms oxygen consumed/min/mg mitochondrial protein.

## 3. Results

### 3.1. Physiological Outcomes

**Figure 1:**
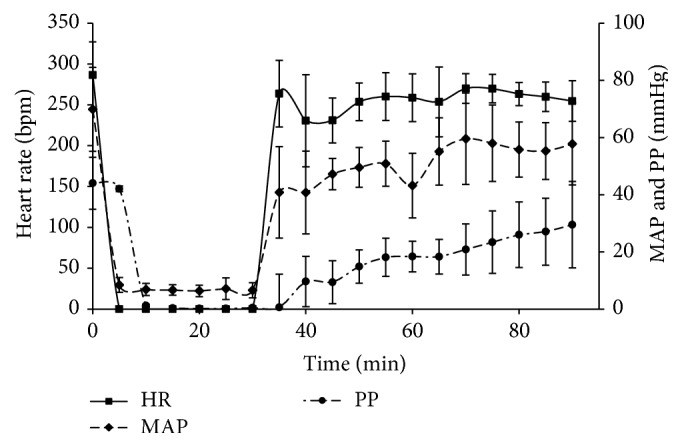
Heart rate (HR), mean arterial pressure (MAP), and pulse pressure (PP) following 30 mim CA and 60 min CPB resuscitation.

Cardiac data on heart function following CA and CPB resuscitation is similar to our previously reported data (Figure 1) [16, 17]. Within 5 min of asphyxia, mean arterial pressure (MAP) fell below 10 mmHg and heart rate (HR) and pulse pressure (PP) became essentially zero. Following 60 min CPB resuscitation, HR, MRP, and PP reached 89, 83, and 76% of the initial rates, respectively. Heart function, however, is sustainable only with CPB support. Return of spontaneous circulation (ROSC) was achieved in ~7 min. As shown previously, rats did not show any corneal reflexes or response to toe pinching. Sporadic urination was observed during 60 min CPB resuscitation. Based on these data, we concluded that the heart was significantly recovered with a moderate functional loss. The brain had no observable function and the kidney had adequate function following resuscitation.

### 3.2. Mitochondrial Isolation Yield

**Figure 2:**
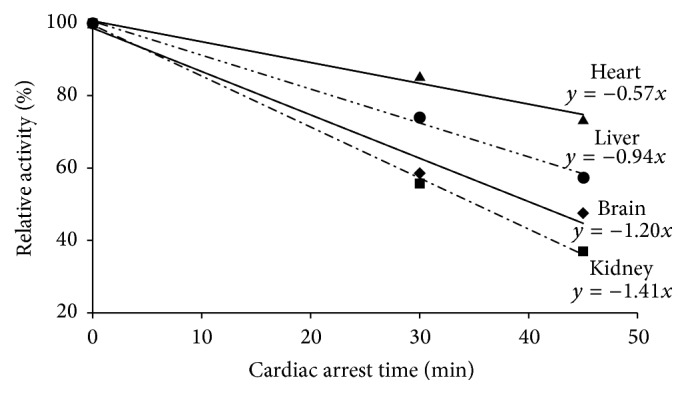
The decrease rates in state-3 respiration of heart, liver, brain, and kidney mitochondria following CA.

**Figure 3:**
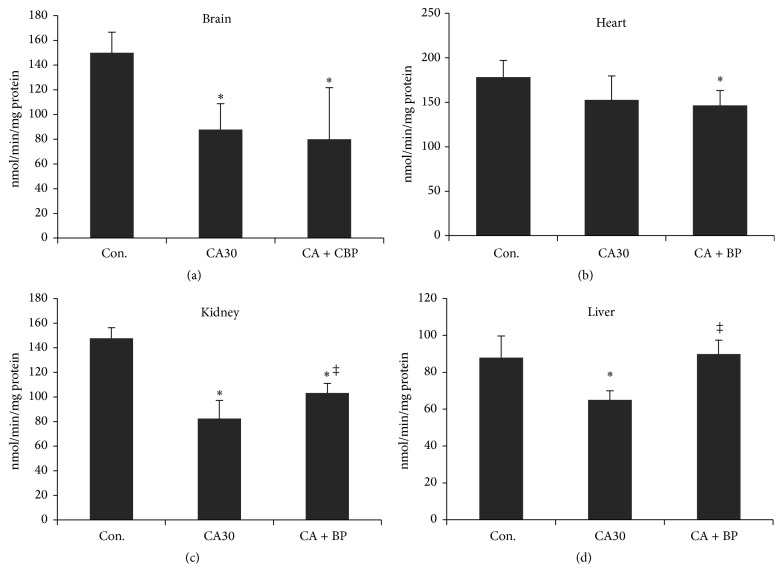
Changes in state-3 respiration activity of mitochondria following 30 min CA or 30 min CA and 60 min CPB resuscitation from brain, heart, kidney, and liver (^*∗*^against  control; ^‡^against  CA30).

**Figure 4:**
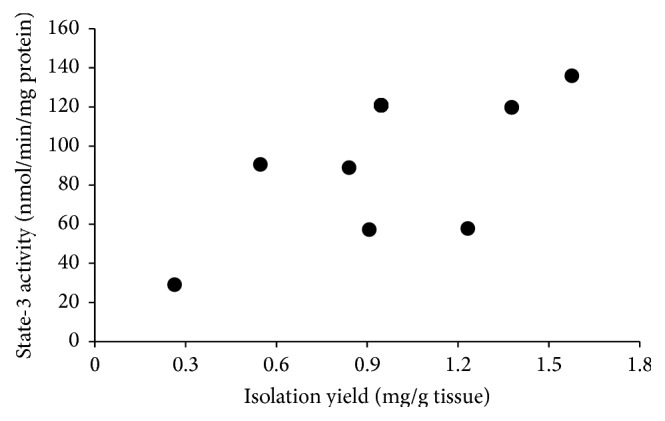
Dot plot of state-3 respiration against isolation yield of brain mitochondria following 30 min CA and 60 min CPB resuscitation.

Mitochondria were isolated.  Table 1 summarizes a complete set of data from examination of isolated mitochondria for this study. Scheme 1 and Figures 1–4 are based on Table 1 to highlight changes in mitochondrial function following extended period of ischemia or CPB resuscitation from 30 min CA. The isolation yields of brain and heart mitochondria were the same between the control and CA groups, suggesting no significant structural decomposition occurred until 45 min CA (Table 1). The yields for kidney and liver mitochondria were decreased following CA. The decrease was from increased tissue weight caused by increased blood content, which was clearly visible in tissue homogenates. In fact, the weight of kidney increased from 1.57 g (control) to 1.83 g (CA30) and 1.84 g (CA45) following CA. The difference in the tissue weight exactly compensates the difference in isolation yield of kidney mitochondria. The decrease in the liver mitochondrial yield was also attributable to increased tissue weight, as the increased blood was observed in the liver homogenates as well following CA. However, brain weight was not increased following CA.

CPB resuscitation following 30 min CA decreased the average brain mitochondrial isolation yield by 40% and the average had a high variability ranging from 0.3 to 1.6 mg per g tissue. The isolation yield of heart mitochondria did not change following CA or resuscitation. The kidney mitochondrial yield was still as low as CA groups due to the same increased tissue weight (1.88 g). Interestingly, the isolation yield of liver mitochondria was increased to the level which is slightly higher than the control following resuscitation.

### 3.3. Mitochondrial Respiration following CA

Following CA, state-3 respiration declined proportionally to the duration of ischemia in mitochondria from all four tissues (Table 1). Compared to their control rates, state-3 respiration activities were decreased by 41% in brain mitochondria, by 15% in heart mitochondria, by 44% in kidney mitochondria, and by 26% in liver mitochondria following 30 min CA. Following 45 min CA, the rates were further decreased by 53, 27, 63, and 43%, respectively. Uncoupled respiration mirrored state-3 respiration in all four mitochondria, suggesting that respirational defect resides in the electron transport chain system. The relative activities of state-3 respiration of mitochondria were plotted against CA time (Figure 2). The response curves for mitochondria from all four tissues were linear with excellent *R* ^2^ values (0.983–0.998), showing that respiration is a good indicator of ischemia. The decrease rates observed in brain and kidney mitochondria were greater than those which occurred in heart and liver mitochondria. This observation is consistent with the notion that the brain and kidney are more vulnerable to ischemia.

Unlike state-3 respiration, state-4 respiration did not change significantly following 30 min CA except for the moderate decrease (20%) found in kidney mitochondria (Table 1). However, the activity in kidney mitochondria returned to the control rate following 45 min CA. State-4 respiration of brain and liver mitochondria did not change following CA for 30 min or 45 min. In heart mitochondria, the activity was increased by 70% only following 45 min CA. Overall, state-4 respiration did not show a correlation with ischemic time and was not a good marker of ischemia.

### 3.4. State-3 Respiration following CPB Resuscitation

We now focus on how state-3 respiration observed following 30 min CA further changes following 60 min CPB resuscitation.  Figure 3 shows that the average state-3 respiration rate remained the same in brain mitochondria (Figure 3(a)). However, the average had a great variability; the activities of mitochondria from two rats ware recovered to the lower control range, whereas the activity of one animal decreased to 20% of the control rate. Since the average mitochondrial isolation yield also had a high variability, we plotted state-3 respiration activity against isolation yield and found a positive correlation between them.

State-3 respiration of heart mitochondria was not changed following CPB resuscitation (Figure 3(b)). Interestingly, state-3 respiration of kidney and liver mitochondria was significantly recovered following CPB resuscitation. Kidney mitochondrial activity was increased to 70% of the control level (Figure 3(c)). Liver mitochondrial activity was completely recovered to the control level following 60 min CPB resuscitation (Figure 3(d)).

## 4. Discussion

### 4.1. Mitochondrial Respiration in Ischemia and Reperfusion

Mitochondrial respiration is the central mechanism for ischemia [18–20] and normalizing mitochondrial respiration is essential for the recovery of organ function as well as successful resuscitation [9, 21]. Particularly, the linear response of the decrease in state-3 respiration to the duration of ischemic time in all four organs (Figure 2) suggests that state-3 respiration is a good indicator of the progression of ischemic damage in each tissue. Therefore, following the activity of state-3 respiration will provide information on the efficacy of resuscitation protocols for each organ.

Another key observation is that mitochondrial isolation yield was not affected by the duration of ischemia. Mitochondrial isolation yields provide information on mitochondrial structural integrity. Any injury models, which cause morphological change, may induce changes in mitochondrial density. These mitochondria will be excluded during the isolation process, resulting in lower isolation yields. In this light, constant mitochondria isolation yields following 45 min CA confirm that ischemia does not cause significant changes in mitochondrial density and that there is no selective loss of damaged mitochondria.

### 4.2. Effect of Reperfusion on State-3 Respiration of Brain Mitochondria

The state-3 respiration activities of brain mitochondria from individual animals vary widely following 60 min CPB resuscitation (Figure 4); the activity in one animal recovers to 90%, while in another animal it drops to 20% of the control activity. In addition, the isolation yields of brain mitochondria from most animals decrease with a high variability. The positive correlation between the activity and the yield (Figure 4) suggests that animals with more activity loss also suffer more from structural alterations of brain mitochondria. Therefore, it seems that activity loss is accompanied by structural alterations following resuscitation and proper perfusion can significantly recover the respiration activity with minimal structural alterations in mitochondria.

The decrease in isolation yield excludes no-reflow phenomenon as the reason for the loss of activity, because 90 min CA does not result in a decreased isolation yield (data not shown). Rather, it suggests that the inconsistent and/or insufficient perfusion is responsible for the inconsistent response of brain mitochondria to resuscitation. In support of this view, previous reports proposed that inadequate perfusion is more harmful than no perfusion [22]. The brain is a heterogeneous tissue with many compartments. A subtle difference in the severity of damage, microvascular obstruction, or edema may interfere with the blood flow to the brain resulting in inconsistent global or local perfusion. Mitochondria in regions with insufficient flow undergo additional damage accompanied by structural alterations.

Another possible explanation may be the existence of a damage threshold. Mitochondria damaged beyond the threshold (irreversible damage) during ischemia are subjected to additional activity loss and/or decomposition during reperfusion. On the other hand, mitochondria with slight damage recover with reperfusion, and, by 30 min CA, mitochondria with both reversible and irreversible damage can be observed in the brain. Overall, the high variability in respiration activity and isolation yield well represent the difficulty and complexity commonly observed in investigating brain perfusion in human patients and animal models [23].

### 4.3. Effect of Reperfusion on State-3 Respiration of Heart Mitochondria

The achievement of ROSC and hemodynamic data show the resumption of heart function following CPB resuscitation. However, the decrease in HR, MRP, and PP also showed that heart function was compromised at some degree (Figure 1). Consistent with this physiological outcome of the heart, the 15% decrease in state-3 respiration, which occurred following CA, still exists following resuscitation. Interestingly, heart mitochondria with the least activity loss following CA do not show any sign of recovery following CPB resuscitation.

Preventing additional decreases in state-3 respiration indicates that reperfusion significantly normalizes preceding ischemic alterations in the heart. However, this normalization is not sufficient to improve state-3 respiration. The reason that mitochondrial respiration remained inhibited is not clear. One possible explanation is that reperfusion may have caused additional damage to mitochondria, as evidenced by the decrease in uncoupled respiration following 60 min resuscitation (Table 1). In any case, simply restoring blood flow will not completely rescue the heart from prolonged ischemia and additional intervention is required.

### 4.4. Effect of Reperfusion on State-3 Respiration of Kidney and Liver Mitochondria

The respiration activities of kidney and liver mitochondria are significantly recovered by CPB resuscitation, suggesting that sufficient perfusion substantially normalizes molecular alterations and metabolic disorders. It is noteworthy that kidney mitochondria lose state-3 activity more than the other mitochondria but still respond well to CPB resuscitation. The remaining 30% inhibition of state-3 respiration again may be caused by permanent ischemic damage or ongoing reperfusion injury. The remained increased tissue weight, which occurred following CA, suggests that ischemic alterations still exist at some degree and contribute to the 30% decrease in activity.

Following 60 min CPB resuscitation, state-3 respiration of liver mitochondria is completely recovered to control level. Mitochondrial isolation yield also becomes normal. The recovery of liver seems to be the most prominent of the four organs by CPB resuscitation. Apparently, no reperfusion injury is evident in the state-3 respiration in liver mitochondria following 60 min CPB resuscitation. The results suggest that the liver is the most resistant to ischemia/reperfusion injury.

Overall, the results show that following state-3 respiration is a useful approach to gain insights into how each tissue responds to ischemia and resuscitation. Time course studies varying the duration of CA and resuscitation will help to better understand how injury severity affects the response of each organ to different resuscitation protocols. In addition, correlation of state-3 respiration with the physiological outcome and survival of animals following resuscitation will elucidate the role of mitochondrial respiration for ischemia/reperfusion injury in CA.

## 5. Conclusion

Using 30 min CA and 60 min CPB resuscitation, we study the effect of ischemia and reperfusion on mitochondrial respiration in the brain, heart, kidney, and liver. State-3 respiration of mitochondria from all four organs decreases proportionally to the duration of ischemic time. The decrease rates are greater in brain and kidney mitochondria than in heart and liver mitochondria. Following 60 min CPB resuscitation, state-3 respiration activity of brain mitochondria varies from animal to animal. This inconsistent response of brain mitochondria well represents the difficulty in studying the response of brain to resuscitation in human patients and animal models. The respiration activity does not change in heart mitochondria and significantly increases in kidney and liver mitochondria. The results suggest that each organ responds differently to CPB resuscitation and this difference should be considered to design better resuscitation protocols.

## Figures and Tables

**Table 1:** Mitochondrial isolation yield (mg/g tissue) and respiration activities (NO/min/mg mitochondrial protein).

	Isolation yield				State-3			
	Con.	CA30	CA45	CA30 + BP60	Con.	CA30	CA45	CA30 + BP60
Brain	1.52 ± 0.22	1.44 ± 0.33	1.61 ± 0.40	0.99 ± 0.41^*∗*^	150 ± 16.6	87.9 ± 20.9^*∗*^	71.2 ± 10.2^*∗*^	87.6 ± 37.3^*∗*^
Heart	20.4 ± 1.1	19.2 ± 2.3	19.9 ± 2.0	18.9 ± 2.4	178.3 ± 18.9	152.0 ± 28.1	124.0 ± 27.2^*∗*^	146.4 ± 16.6^*∗*^
Kidney	17.8 ± 2.8	14.6 ± 1.3^*∗*^	15.3 ± 1.0	15.1 ± 1.9	147.7 ± 8.7	82.4 ± 14.8^*∗*^	54.7 ± 15.5^*∗*‡^	103.3 ± 7.8^*∗*‡^
Liver	22.7 ± 2.4	16.3 ± 1.8^*∗*^	19.2 ± 2.2^*∗*^	26.6 ± 3.4^‡^	87.9 ± 11.7	65.1 ± 4.9^*∗*^	50.4 ± 6.9^*∗*‡^	89.8 ± 7.6^‡^

	State-4				Uncoupled			
	Con.	CA30	CA45	CA30 + BP60	Con.	CA30	CA45	CA30 + BP60

Brain	21.4 ± 5.5	19.6 ± 6.0	20.9 ± 3.4	18.7 ± 4.6	166.3 ± 24.5	93.0 ± 29.2^*∗*^	74.6 ± 11.4^*∗*^	101.4 ± 54.8^*∗*^
Heart	21.4 ± 5.2	23.7 ± 6.0	35.8 ± 8.3^*∗*‡^	19.7 ± 4.1	182.8 ± 32.7	171.7 ± 21.6	140.0 ± 37.2	144.0 ± 17.4
Kidney	20.6 ± 2.8	16.3 ± 4.6	18.7 ± 5.0	15.4 ± 2.7	162.3 ± 10.3	91.1 ± 24.5^*∗*^	56.7 ± 20.7^*∗*‡^	115.0 ± 15.5^*∗*^
Liver	12.0 ± 3.1	11.9 ± 3.8	14.5 ± 2.4	12.5 ± 3.8	120.3 ± 22.2	97.9 ± 8.6^*∗*^	78.0 ± 12.0^*∗*^	127.1 ± 9.3^‡^

^*∗*^Against control; ^‡^against CA30.
